# Judgments of effort for magical violations of intuitive physics

**DOI:** 10.1371/journal.pone.0217513

**Published:** 2019-05-23

**Authors:** John McCoy, Tomer Ullman

**Affiliations:** 1 The Wharton School, University of Pennsylvania, Philadelphia, United States of America; 2 Department of Psychology, Harvard University, Cambridge, United States of America; Middlesex University, UNITED KINGDOM

## Abstract

People spend much of their time in imaginary worlds, and have beliefs about the events that are likely in those worlds, and the laws that govern them. Such beliefs are likely affected by people’s intuitive theories of the real world. In three studies, people judged the effort required to cast spells that cause physical violations. People ranked the actions of spells congruently with intuitive physics. For example, people judge that it requires more effort to conjure up a frog than to levitate it one foot off the ground. A second study manipulated the target and extent of the spells, and demonstrated with a continuous measure that people are sensitive to this manipulation even between participants. A pre-registered third study replicated the results of Study 2. These results suggest that people’s intuitive theories partly account for how they think about imaginary worlds.

## Introduction

Both children and adults spend much time engaged with make-believe worlds: children pretend, adults daydream, and both immerse themselves in movies and novels. But even in make-believe, not everything goes. Superman leaps tall buildings in a single bound, but a building takes more sweat than an ant-hill. And even for Superman, leaping to Alpha Centauri is simply silly.

Adults and children have a nuanced understanding of fictional worlds, for example they understand that there are multiple fictional worlds, and that the characters in these worlds believe this too [1,2]. People rely on their knowledge of the real world when they create such imaginary worlds. For example, when people generate imaginary animals, they do not differ much from those found on Earth [3,4,5], and people believe that real world facts hold true for different fictional worlds depend on the type of fact and the distance of the narrative world from the real world [6]. People have graded judgments of impossibility in fictional worlds, as Shtulman and Morgan [7] demonstrated by having people judge the difficulty of spells supposedly from the Harry Potter curriculum. They further argued that people draw on subsidiary principles to judge degrees of impossibility when a central causal principle is violated. More generally, in her book “The Rational Imagination”, Byrne [8] outlines a set of principles that govern the possibilities that people consider when reasoning about both reality and counterfactual alternatives.

Previous research has suggested that people’s knowledge of many real world domains takes the form of an intuitive theory [9,10,11]. Like scientific theories, intuitive theories organize a domain in terms of a set of concepts, and rules that govern how the concepts interact. As with a scientific theory, an intuitive theory can be used to predict, explain, compress, and interpret data [12,13,14]. Computationally, an intuitive theory can be captured by a probabilistic generative model of a domain [15].

One important intuitive theory is intuitive physics, which organizes people’s understanding of the behavior of everyday objects. Some physical expectations, such as permanence and cohesion, are innate or learned extremely early [16,17], while others develop over the first years of life [18,19]. Intuitive physics is also an important aspect of adult scene-understanding, guiding people as they predict, infer and take action in dynamic environments, e.g. [20–23]. A promising formal model of people’s intuitive physics is the mental physics engine, analogous to the software underlying modern video games, and see e.g. [24]. Given the fundamental role of intuitive theories, they may be an important part of how we imagine fictional worlds.

People’s intuitions about fictional worlds can be used to reveal people’s intuitions about the real world. For example, Kelly and Keil [25] examined what transformations occur in myths and folklore as a window into how people organize concepts. More recently, Griffiths [26] investigated people’s ontological commitments by having participants judge the relative interest of different stage-magic tricks.

While previous research has shown that people rely on their knowledge of the real world to construct imaginary worlds, we suggest that one important way that people do so is by drawing on their intuitive theories. When people judge the plausibility of a particular physical violation in a magical world, they may consider the amount of force or effort that their intuitive theory of physics suggests would be needed to bring that change about in the real world. We focus on the domain of intuitive physics, and present three studies in which people estimate the effort required to cast spells that cause physical violations. In Study 1, participants ranked ten spells by effort, each of which caused a physical violation such as levitation or petrification. Study 2 used a continuous measure of effort to examine the same spells, but also varied the target of each spell between conditions (levitating a frog or levitating a cow). If people indeed rely on a shared intuitive physical theory, their judgments are likely to be consistent within and across conditions, and sensitive to changes in the target or extent of the spells in line with intuitive physics. We found there was indeed consistency both between and across conditions as to which spells were judged more or less effortful. Participants’ rankings were unaffected by the amount of exposure participants had to fantasy and magic in media. In a pre-registered third study, we replicate the results of Study 2.

## Study 1

### Methods

Participants (N = 201) were recruited from Amazon’s Mechanical Turk service, restricted to people in the United States, with an approval rating of 95% or above and more than 500 approved HITs. Participants were compensated for their participation. The sample size was chosen in advance, given an expectation of medium effect sizes. The data were not analyzed until all participants had responded. Six participants were excluded for failing an attention check, as explained below. The age of the participants ranged from 18 to 83, with a median age of 31 years. Of the participants, 72 identified as female. No additional demographic information was collected from participants. Participants provided informed consent via web-form, for this and the next studies. All experiments were approved by the MIT Committee on the Use of Experiments with Human as Experiment Subjects.

Participants were asked to imagine a world in which wizards cast spells. Each spell required magic points, reflecting the effort needed to cast that spell. Ten different spells (middle column of Table 1) were chosen to examine different physical violations, all targeting the same object. Participants ranked the spells by how effortful they estimated the spells were to cast. The spells were presented as a list in random order, and participants ranked the spells by dragging them one at a time to a new position on the screen, such that they formed an ordered list (Materials, analysis code, and data for all studies are available here: https://osf.io/75qkg/)

**Table 1:** Different spells used in Study 1, and conditions A and B of Studies 2 and 3.

Spell	Studies 1, 2A, and 3A	Studies 2B and 3B
Conjure	Conjure a frog into existence	Conjure a cow into existence
Invisible	Turn a frog invisible	Turn a cow invisible
Teleport	Teleport a frog 1 foot forward	Teleporting a frog 100 feet forward
Cease	Make a frog cease to exist	Make a cow cease to exist
Color	Change a frog's color to purple	Change a frog’s color to purple
Levitate	Levitate a frog 1 foot off the ground	Levitate a frog 100 feet off the ground
Big	Make a frog twice as big	Make a frog 200 times as big
Transform	Turn a frog into a mouse	Turn a frog into a cow
Stone	Turn a frog into a stone frog	Turn a cow into a stone cow
Split	Split a frog into two smaller frogs	Split a frog into 200 small frogs

As an attention check, participants were asked which spell they thought was easiest to cast. Six participants did not respond with their top or bottom ranked spell and were excluded. Responses to this question were also used to determine whether a participant had ranked spells from easiest to hardest or from hardest to easiest. On the next screen, participants indicated how much exposure they had to fantasy and magic in the following media: ‘books and literature’, ‘television and movies’, and ‘games, such as board games and computer games’. Participants used sliders that ranged from 0 (“very small exposure”) to 100 (“very large exposure”). In this study and the other studies reported in the paper, participants completed the task in 3–4 minutes on average, and were paid $0.50 for their time.

### Results

**Fig 1:**
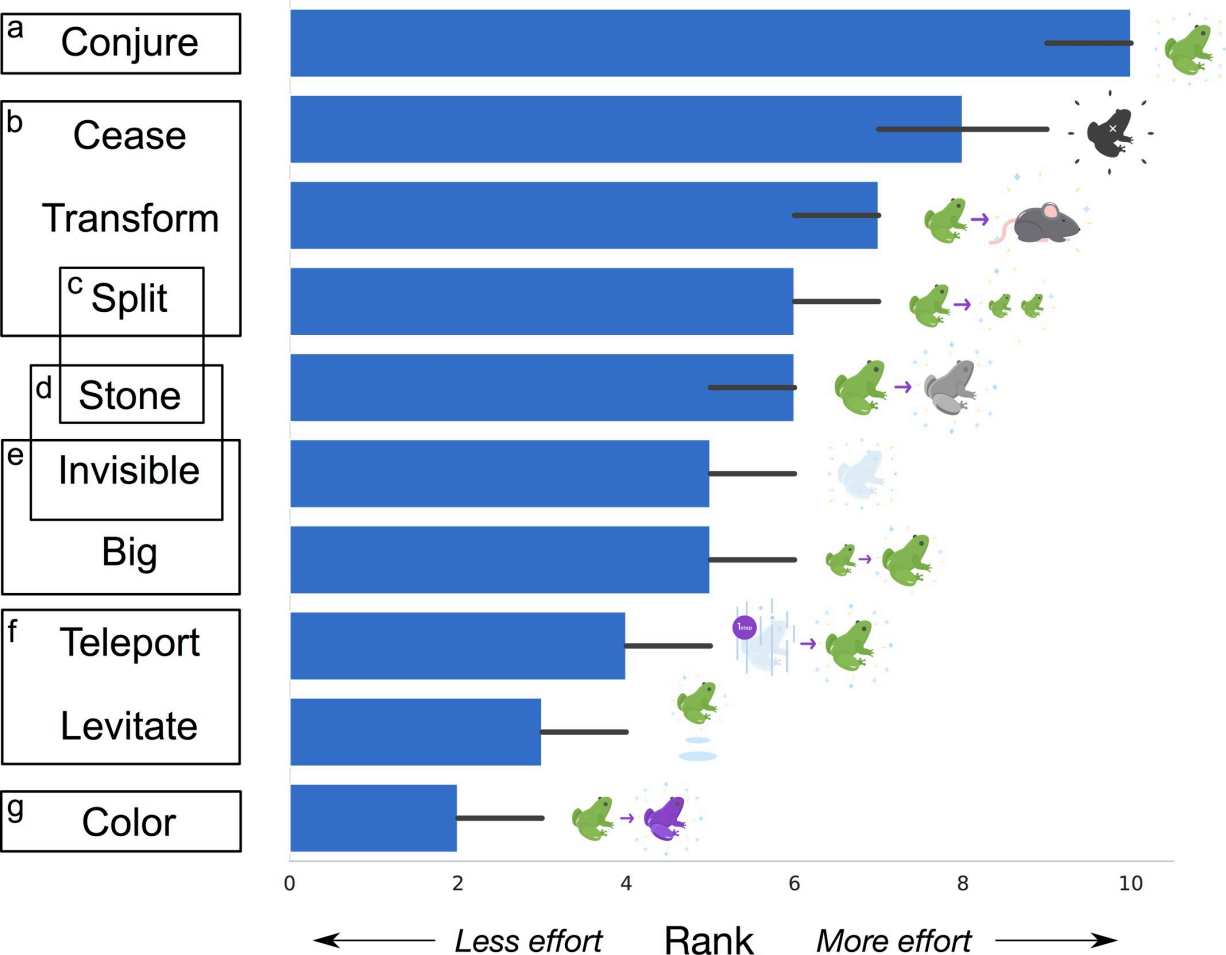
Median ranks for spells in Study 1, with bootstrapped 95% confidence intervals. Spells grouped in the same box and marked with the same letter were not significantly different, by a Wilcoxon signed rank test at the *p = 0*.*05* level. Confidence intervals for a particular spell sometimes do not extend either above or below the median rank for the spell since the summary statistic being used is medians. Spell labels correspond to the descriptions found in Table 1.

Each of the ten spells was ranked by each participant from rank 1 (easiest spell) to rank 10 (hardest spell). Fig 1 shows the median rank of the different spells across participants, with 95% bootstrapped confidence intervals. Participant rankings for every pair of spells were compared with a Wilcoxon signed rank test, as shown in Fig 1. Across participants *Conjure* was ranked as the hardest spell, *Color* was ranked as the easiest, and the remaining spells fall into different groups in between.

**Fig 2:**
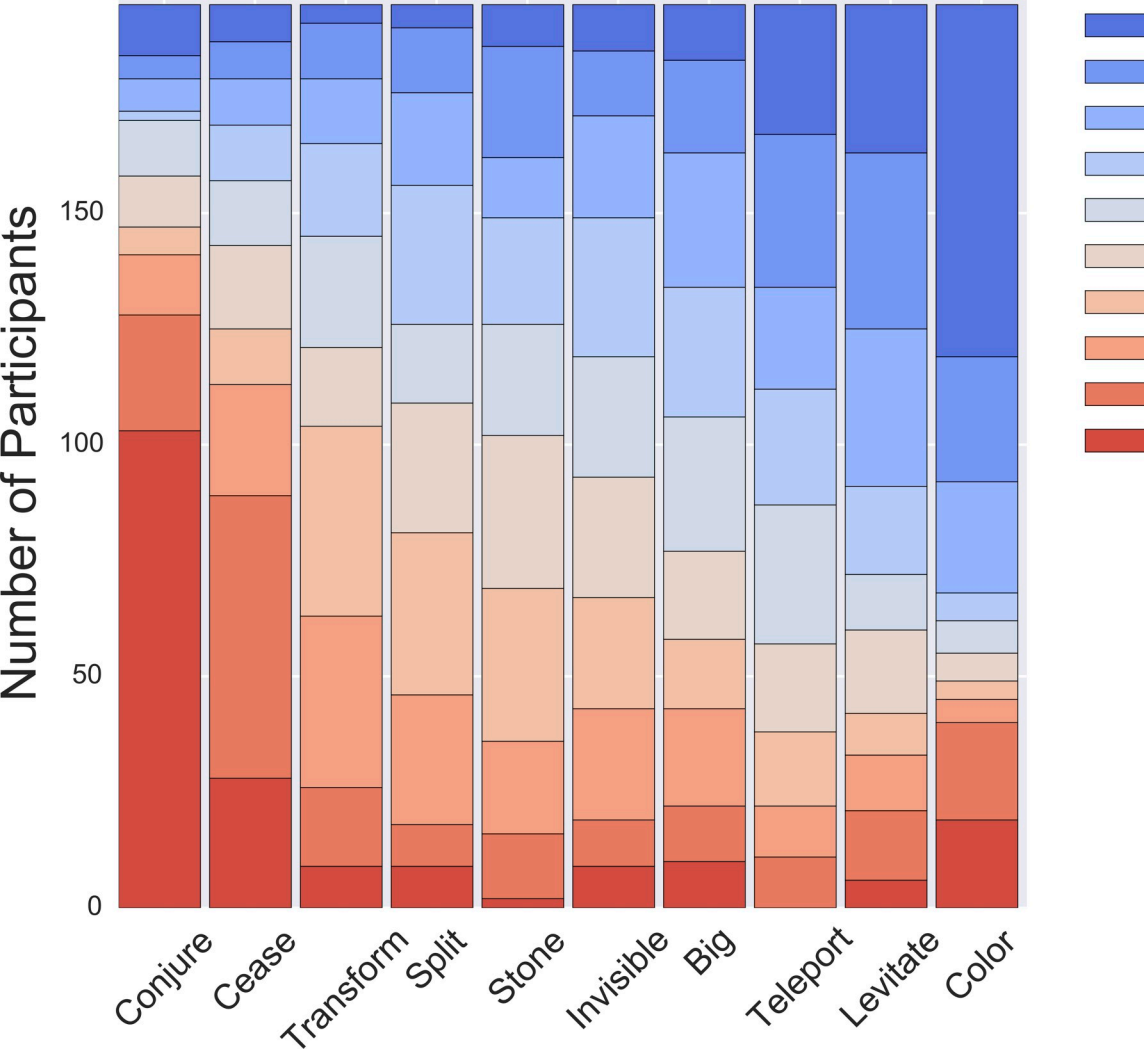
Distribution of the ranks for each spell in Study 1. Each column shows how many participants gave each rank for that spell. For example, 103 participants (53%) gave a rank of 10 to *Conjure*, 28 participants (14%) gave it a rank of 9, and so on.

We next examined how much variance exists between participants, shown in Fig 2 as the distribution of ranks for each spell. To determine how much individual participants agreed with the median ranking, we computed the correlation between each participant’s own ranking and the median ranking (*r*_*S*_ = 0.54, *SE = 0*.*07*, after an *r-Z-r* transformation).

A median-split analysis on the amount of exposure to fantasy in different media showed no relationship between amount of exposure and spell rankings. The correlation between the spell rankings of the high and low exposure groups was r_*S*_ = 0.96 for movies, r_*S*_ = 0.97 for books, r_*S*_ = 0.97 for games, and r_*S*_ = 0.98 when summing exposure across all media (Participants reported an average exposure to fantasy and magic of 61 (*SD = 30*) for movies, 53 (*SD = 33*) for books, and 57 (*SD = 36*) for games).

## Study 2

In Study 1, participants showed a consistent ranking of spell difficulty, from changing color (easiest) to conjuring (hardest), and exposure to fantasy had no effect on how participants ranked the spells. Study 2 tests the effect of varying the target of each spell between participants while keeping the spell itself fixed (for example, levitating a frog vs. levitating a cow). Participants provided a continuous measure of spell difficulty, rather than giving rank judgments.

### Methods

A new group of participants (N = 370) was recruited via Amazon’s Mechanical Turk service, restricted to people in the United States who did not participate in Study 1, with an approval rating of 95% or above and more than 500 approved HITs. Participants were compensated for their participation. We excluded 30 participants who failed attention checks, as explained below. The age of the remaining participants ranged from 18 to 69, with a median age of 32 years. Of the participants, 137 identified as female. No additional demographic information was collected from participants.

As in Study 1, participants were asked to imagine a world in which wizards cast spells, with each spell requiring magic points that reflect the effort needed to cast that spell. In order to interpret all points relative to a common baseline, participants in both conditions were told that changing a frog's color to purple (the spell judged as easiest in Study 1), requires ten magic points. Ten points was chosen as a baseline to easily allow participants to give spells fewer points than this if they did not believe that changing color was the easiest spell.

Participants were shown a list of spells in randomized order, and judged the number of magic points each spell required by entering a number into a text field. Participants were randomly assigned to one of two conditions. In Condition A (N = 158), the spells were the same as in Study 1. In Condition B (N = 182), each spell was the same as its counterpart in Condition A, but either the target or extent of the spell was different. If the spell in condition A explicitly contained a number, then this number was multiplied by 100 to produce a counterpart spell for condition B (for example, levitating a frog 100 feet rather than 1 foot). Otherwise, the target of the spell was changed from a frog to a cow (for example, conjuring a cow rather than a frog). See Table 1 for the full list of spells used in both conditions.

After estimating the effort required for the spells, participants were presented with two attention checks. One attention check asked participants to report which spell, from the list that they saw, required the most magic points. A second question asked how many magic points were required to change a frog’s color to purple. As in Study 1, participants indicated how much exposure they had to fantasy and magic in different media by using sliders that ranged from 0 (“very small exposure”) to 100 (“very large exposure”).

### Results

We first consider the rank ordering of the spells. For each participant, we converted the points they assigned to the spells to a rank ordering of spells. For example, if a participant assigned fewer points to *Levitate* than any other spell, then for that participant *Levitate* has a rank of 1. Following this conversion, we ordered the spells in each condition by median rank across participants, allowing for a comparison with the rank ordering found in Study 1. As summarized in Table 2, we found that the rank ordering of the spells in Condition A and Study 1 agree (*r*_*S*_ *= 0*.*97*), thus replicating the results of Study 1. We also compared the spell ordering for different spell targets: the rank ordering of the spells in Condition A and Condition B agree (*r*_*S*_ *= 0*.*94*), and there was agreement between Study 1 and Condition B (*r*_*S*_ *= 0*.*93*).

**Table 2:** Spearman correlations between median ranks in Studies 1–3, after point responses in Studies 2–3 are turned into ranks per participant.

	Study 1	Study 2A	Study 2B	Study 3A	Study 3B
Study 1	1.00	0.97	0.93	0.95	0.93
Study 2A		1.00	0.94	0.99	0.91
Study 2B			1.00	0.94	0.99
Study 3A				1.00	0.91
Study 3B					1.00

We further examined the relative difficulty of the spells by ordering them according to the median number of points assigned to each spell across participants. Such an analysis, summarized in Table 3, again showed high agreement between Condition A and Study 1 (*r*_*S*_ *= 0*.*97*), Condition B and Study 1 (*r*_*S*_ *= 0*.*89*), and Condition A and Condition B (*r*_*S*_ *= 0*.*96*).

**Table 3:** Spearman correlations between participants’ median ranks (Studies 1–3), after transforming median point responses in Studies 2 and 3 into ranks.

	Study 1	Study 2A	Study 2B	Study 3A	Study 3B
Study 1	1.00	0.97	0.89	0.93	0.97
Study 2A		1.00	0.96	0.98	0.99
Study 2B			1.00	0.94	0.95
Study 3A				1.00	0.98
Study 3B					1.00

**Fig 3:**
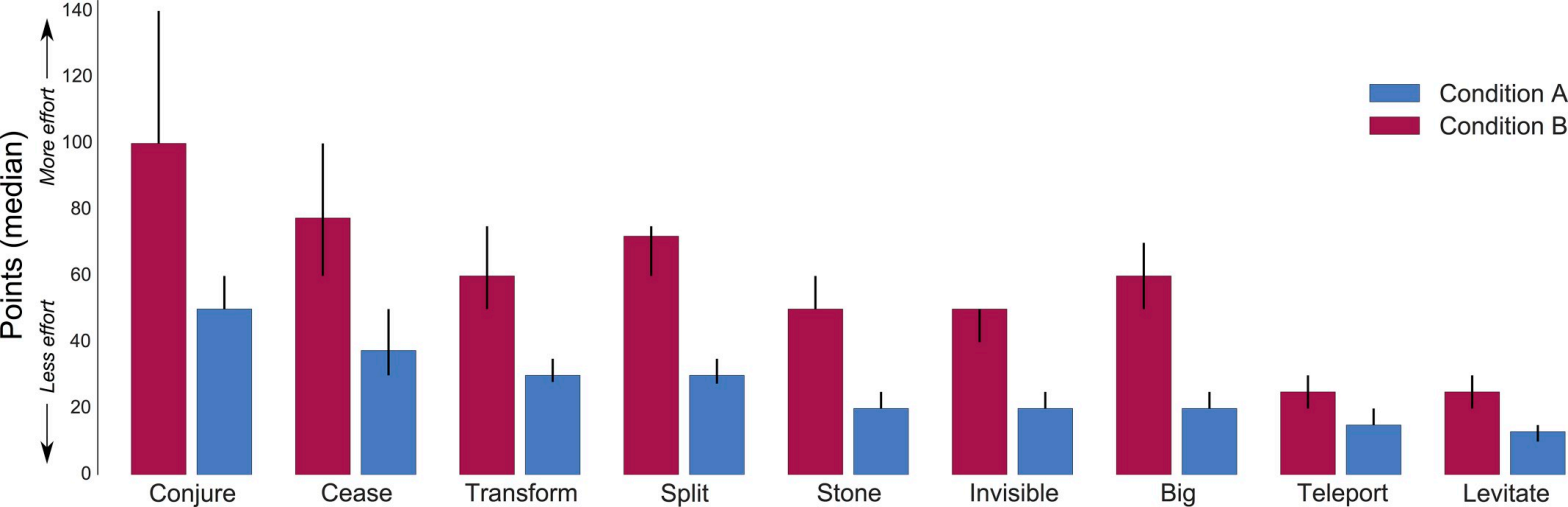
Median points assigned to each spell in the two conditions of Study 2. Error bars are bootstrapped 95% confidence intervals. Spell labels correspond to the descriptions in Table 1. As discussed in the text, participants in both conditions were told that the spell to change a frog's color to purple requires ten magic points.

The continuous points participants assigned to spells allow an analysis of perceived spell difficulty beyond their relative ordering. Fig 3 shows the median number of points assigned to each spell, both within condition (the spells vary, e.g. levitating versus conjuring) and between conditions (the target of the spells vary, e.g. levitating a frog versus levitating a cow). Fig 3 displays the median point judgments for each spell. Because participants were not constrained to a pre-specified range for their point judgments, there was a large amount of individual variability. Within conditions, Fig 3 shows the ranked difficulty of the spells, and the magnitude of the differences in effort between spells. For example, *Conjure* was about 4 times more effortful than *Levitate* in both conditions. Condition B spells were judged as harder than their counterparts in Condition A: for every spell the median points assigned in Condition B was higher than its counterpart in Condition A, with non-overlapping 95% confidence intervals. Note that the difficulty of the spells may have changed in multiple ways between conditions A and B: both because some spells changed their target and others their extent, but also because changing the target or extent may have differential impact depending on the spell. Thus, while we found that the spells had similar rank orders across the two conditions this need not have been the case, and similarly the ratio of points across spells need not have been fairly constant across conditions. In the General Discussion, we consider how varying different aspects of a spell may change its difficulty.

We assessed agreement among participants by computing the correlation between each participant’s ranking and the median ranking. This resulted in an average correlation of *r*_*S*_ *= 0*.*66* (*SE = 0*.*08*) for Condition A, and *r*_*S*_ *= 0*.*69* (*SE = 0*.*07*) for Condition B, both after an *r-Z-r* transformation.

A median-split analysis on the amount of exposure to fantasy in different media showed no relationship between amount of exposure and spell rankings for either condition in Study 2. The correlation between the spell rankings of the high and low exposure groups for movies was r_*S*_ = 0.97 (Condition A) and r_*S*_ = 0.91 (condition B), for books was r_*S*_ = 0.96 (Condition A) and r_*S*_ = 0.94 (Condition B), for games was r_*S*_ = 0.94 (Condition A) and r_*S*_ = 0.84 (Condition B), and when summing exposure across all media was r_*S*_ = 1.0 (Condition A) and r_*S*_ = 0.9 (Condition B). Participants reported an average exposure of 64 (*SD = 27*) for movies, 54 (*SD = 32*) for books, and 58 (*SD = 35*) for games.

## Study 3

To heighten confidence in the results of the first two studies, we ran a third study with identical methods and stimuli as in Study 2, with pre-registered methods and analysis, and a larger sample size. Pre-registration details and materials can be found in an OSF repository at https://osf.io/75qkg, under ‘registrations’.

### Methods

Participants (N = 600) were recruited from Amazon’s Mechanical Turk service, restricted to people who had not participated in the previous studies, with an approval rating of 97% or above, and more than 500 approved HITs. Participants were compensated for their participation. After restricting to US participants, 39 participants were excluded. A further 81 participants were excluded for failing attention checks, as in Study 2. The age of the remaining participants (N = 479) ranged from 18 to 70, with a median age of 34 years. Of the participants, 122 identified as female. Study 3 used the same survey as Study 2, with the same conditions A (N = 241) and B (N = 238).

### Results

We computed the rank ordering of spells as in Study 2, and found agreement between the rank ordering of spells in Study 2 and Study 3, summarized in Table 2. All correlations between and within the different conditions of Studies 2 and 3, as well as with Study 1, were high (0.91 and above). If the spells in Study 3 are ordered by the median number of points assigned to each spell, there is again high agreement between studies and within conditions (summarized in Table 3).

As in Study 2, spells in Condition B were judged as harder than their counterparts in Condition A: the median number of points assigned to every spell in Condition B was higher than its counterpart in Condition A, with non-overlapping 95% confidence intervals.

We again assessed agreement among participants by computing the Spearman correlation between each participant’s ranking and the median ranking. This resulted in an average correlation of r_S_ = 0.67. (SE = 0.06) for Condition A, and r_S_ = 0.64 (SE = 0.07) for Condition B, both after an *r-Z-r* transformation. This is similar to the level of agreement found in Study 2.

As in Study 2, a median-split analysis on the amount of exposure to fantasy in different media showed no relationship between amount of exposure and spell rankings for either condition. The correlation between the spell rankings of the high and low exposure groups for movies was r_*S*_ = 0.99 (Condition A) and 0.93 (Condition B), for books was r_*S*_ = 0.99 (Condition A) and 0.98 (Condition B), for games was r_*S*_ = 0.99 (Condition A) and r_*S*_ = 0.92 (Condition B), and when summing exposure across all media was r_*S*_ = 0.98 (Condition A) and r_*S*_ = 0.9 (Condition B). Participants in Study 3 reported an average exposure of 65.7 (SD = 25.9) for movies, 54.5 (SD = 30.4) for books, and 56.2 (SD = 33.8) for games, similar to Study 2.

Study 3 thus closely replicates the results of Study 2 for every pre-registered analysis, increasing confidence in the results presented here.

## General discussion

Sadly, magic does not exist, but how do people think about worlds that have magic in them? More generally, how do people understand or construct imaginary worlds? We suggest that people draw on their intuitive theories of the real world to reason about imaginary worlds. In three studies, we presented people with spells that violated various physical principles and had them judge how much effort each spell required. Across the three studies, participants were consistent in how much effort they thought the different spells required. The target or extent of a spell (levitating 1 foot versus 100 feet) also affected how effortful a spell was judged. This is particularly striking given that participants saw only a single version of each spell. Exposure to fantasy or magic in the media had no effect on how much effort participants thought that different spells required.

Given that more fundamental principles of intuitive physics are thought to be acquired earlier in development [17], it is interesting to analyze what principle of intuitive physics each spell violates, and to compare the age at which this principle is acquired with the perceived effortfulness of the corresponding spell. The spells perceived as most effortful—*Conjure* and *Cease*—violate object permanence and cohesion, which are the earliest developing principles at the core of object understanding [17,27]. Object permanence and cohesion apply to all objects, and indeed govern why we perceive something as an [16]. The next most effortful spells—*Transform* and *Split—*do not violate object permanence, but they do violate basic principles of kind-identity and numerosity. Infants and children perceive certain features as essential to the kind-identity of an object [28,29], and expect a particular object to neither change its kind [30] nor numerosity [31]. These are also early-developing intuitions, though later in appearance than object permanence. Less is known about the development of intuitions regarding transformations that leave objects relatively cohesive, such as those of *Invisible*, *Stone* and *Big*. It would be interesting to test whether young children are surprised by transformations that cause objects to change in size or material properties, and whether such surprise occurs only after they have acquired principles of kind-identity and numerosity. Finally, beyond the existence and essential features of an object, there are accidental features that are true of an object at a given moment while not being essential to it. The location of an object one meter to the right or left makes no difference to its kind identity. The easiest spells—*teleport*, *levitate*, and *color—*change only accidental object properties such as location and color.

In this paper we manipulated only one dimension for each spell, such as height in the case of levitation. It is likely, however, that people are sensitive to multiple dimensions when thinking about physical violations. For example, consider how varying the surface area, mass, opaqueness, intricacy, or color of an object might affect the difficulty of casting an invisibility, levitation, or conjuring spell. Mass probably matters for levitation, but color probably does not. Conjuring a charcoal-black espresso machine into existence might take more effort than conjuring up an equally large, equally black, equally opaque chunk of charcoal.

The spells considered in this paper focused on violations of intuitive physics. However, many possible spells rely on people’s intuitive understanding of other domains. For example, in the domain of psychology it is likely harder to magically cause a large change of preference or belief than a small one (and indeed Morgan and Shtulman [7] find graded notions of impossibility for the domains of psychology and biology). One might imagine that a spell to change a preference from strawberry to blueberry jam is easier than a spell to make someone root for a hated sports team. We conjecture that people’s judgments about spell difficulty in a particular domain depend on their intuitive theory for the domain.

An alternative account of how people understand the workings of imaginary worlds posits that people acquire their views through cultural learning, such as the portrayal of fictional worlds in various media. By this account, the ranking of spell difficulty might be based on how magic is portrayed in books or television or games, such as *Harry Potter* or *Lord of the Rings*. Cultural norms may influence judgements of the difficulty of various magical violations, especially in domains where intuitive theories are shaped by cultural knowledge, such as intuitive psychology, sociology, and biology. A limitation of our studies is that they were carried out with US-based participants, and future work could examine judgements of effort for magical violations with people from different cultural backgrounds. Still, people’s ranking of the spells in all our studies were not affected by exposure to fantasy and magic in the media. We suggest that the media does not primarily affect what spells are seen as more difficult, but rather that people bring their intuitive physics to bear when they engage with fiction. That is, in line with previous research on myths and transformation [25], systems of magic are perceived as coherent to the extent to which they match people’s intuitive theories. People perceive levitating a frog as easy not because they know it’s one of the first charms that any young wizard learns at Hogwarts, rather young wizards learn that spell first because readers expect that spell should be easy.

In his 1893 essay *The Fantastic Imagination*, the novelist George Macdonald wrote, “The natural world has its laws, and no man must interfere with them …but they themselves may suggest laws of other kinds, and man may, if he pleases, invent a little world of his own”. It seems people’s little worlds do not stray far from home.
